# Development and evaluation of near-isogenic lines for brown planthopper resistance in rice cv. 9311

**DOI:** 10.1038/srep38159

**Published:** 2016-11-30

**Authors:** Cong Xiao, Jie Hu, Yi-Ting Ao, Ming-Xing Cheng, Guan-Jun Gao, Qing-Lu Zhang, Guang-Cun He, Yu-Qing He

**Affiliations:** 1 National Key Laboratory of Crop Genetic Improvement, Huazhong Agricultural University, Wuhan 430070, China; 2 State Key Laboratory of Hybrid Rice, College of Life Sciences, Wuhan University, Wuhan 430070, China

## Abstract

Brown planthopper (BPH) is the most destructive pest of rice in Asia. To date 29 BPH resistance genes have been identified, but only a few genes are being used in breeding due to inefficient markers for marker-assisted selection (MAS) and little knowledge of the real effects of the genes. In this study we individually transferred 13 genes or QTLs (*Bph14*, *QBph3*, *QBph4*, *Bph17*, *Bph15*, *Bph20*, *Bph24*, *Bph6*, *Bph3*, *Bph9*, *Bph10*, *Bph18* and *Bph21*) into cultivar 9311 by marker assisted backcross breeding (MABB). Through positive and negative selection we narrowed the segments from donors containing *Bph14*, *Bph15*, *Bph6* and *Bph9* to 100–400 kb. Whole-genome background selection based on a high resolution SNP array was performed to maximize reconstitution of the recurrent parent genome (RPG 99.2–99.9%). All genes reduced BPH growth and development and showed antibiotic responses in seedlings. Based on genetic effects and amino acid sequences of genes in three clusters we inferred that *Bph10* and *Bph21* might be identical to *Bph26*, whereas *Bph9* and *Bph18* were different. *Bph15* might be same with *Bph17*, but *QBph4*, *Bph20* and *Bph24* might be different. We believe that these NILs will be useful in rice BPH resistance research and breeding.

Brown planthopper (BPH; *Nilaparvata lugens* Stål), a monophagous sucking insect, is the most damaging insect pest of rice in Asia^1^,^2^. After BPH feeding at the tiller base host plants generally appear yellow and withered^3^, with heavy infestation causing “hopperburn”^4^,^5^. BPH also transmits viral pathogens such as grassy stunt virus (RGSV) and ragged stunt virus (RRSV)^6^. Conventional methods of controlling BPH depend on insecticides that are costly in terms of labor, money, and the environment. Furthermore, overuse of insecticides may affect populations of natural enemies of BPH and lead to resistance/tolerance to insecticides, and resurgence of the BPH problem^7^. Therefore, breeding for host resistance is considered the most economical and environmentally friendly strategy for BPH control^8^.

At least 29 resistance genes have been identified in *Oryza sativa* spp. *indica* and wild rice species^9^. Among them, 14 genes have been fine-mapped to specific regions on chromosomes 3, 4, 6 and 12^10^ and five genes were cloned. Cloned genes *Bph14*, *Bph18* and *Bph26* (or *bph2*) encode coiled-coil, nucleotide-binding, leucine-rich repeat (CC-NB-LRR) proteins, whereas *Bph17* consists of three clustered genes encoding lectin-receptor kinases, and *Bph29* encodes a B3 DNA-binding domain^8^,^11^,^12^,^13^,^14^.

Molecular marker-assisted backcross breeding (MABB) has greatly improved the efficiency and effectiveness of rice breeding. There are three aspects of MABB, namely, positive selection for the target gene using linked markers, negative selection of alleles from the donor parent surrounding the target gene, and background selection for the maximum recovery of the recurrent parent genome using polymorphic markers covering the whole genome^15^,^16^. MABB has been widely used in rice breeding for disease and insect resistance^16^,^17^,^18^. Likewise, MABB has been used to develop multiple BPH-resistant introgression lines (ILs) and near-isogenic lines (NILs). Linkage drag between *Bph3* and *Wx*^a^ alleles was successfully broken by negative selection allowing development of ILs with broad-spectrum resistance to BPH and good quality^19^. By using marker 7312. T4A that co-segregated with *Bph18* in positive selection, and 260 SSR markers for background selection a group of ILs carrying *Bph18* were developed^20^. However, only a few genes have been exploited successfully in breeding for BPH resistance due to inefficient markers and inadequate knowledge of the actual effects of the resistance genes.

In our study, 13 BPH resistance genes from nine donors [RH (*Bph3* and *Bph17*), B5 (*Bph14* and *Bph15*), IR54751-1-2-44 (*QBph3* and *QBph4*), IR65482-4-136 (*Bph10*), IR71033-121 (*Bph20* and *Bph21*), IR65482-7-216 (*Bph18*), Swarnalata (*Bph6*), Pokkali (*Bph9*) and BR96 (*Bph24*)]^11^,^21^,^22^,^23^,^24^,^25^,^26^,^27^,^28^,^29^ were individually incorporated into cultivar (cv.) 9311 (an elite restorer parent for hybrids in China) by MABB, and 13 monogenic NILs were developed with enhanced BPH resistance. Some NILs carry chromosomal fragments from the donor parents of less than 100 kb, and have reconstituted recurrent parent genomes (RPG) exceeding 99.5% as a result of using a breeding chip with high-density SNP markers for negative and background selection. The conserved amino acid sequences of gene clusters on chromosomes 3, 4 and 12 were compared with those of *Bph14*, *Bph17* and *Bph26*, respectively.

## Results

### Development of monogenic NILs

**Figure 1:**
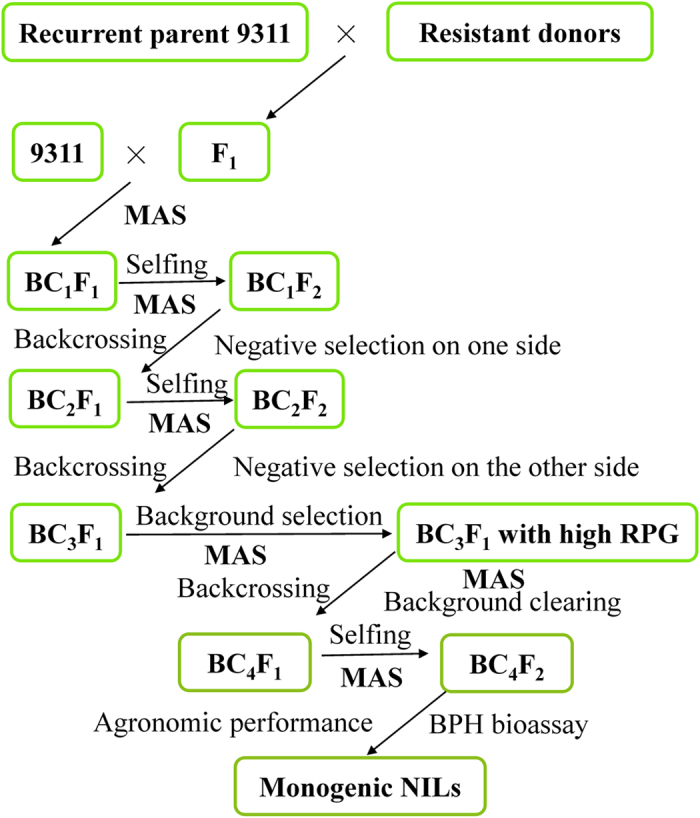
Strategy used to develop NILs. The resistance donors included 9 cultivars: RH, B5, IR54751-1-2-44, IR65482-4-136, IR71033-121, IR65482-7-216, Swarnalata, Pokkali and BR96.

To develop monogenic NILs, 13 BPH resistance genes or QTLs, identified in nine donor accessions were individually incorporated into 9311 by MABB (Fig. 1). The entire scheme took 9 crosses, 4 generations of backcrossing and one generation of selfing. In each backcross generation, individuals heterozygous at the target locus were further backcrossed to the recurrent parent 9311.

### Positive and negative selection

**Figure 2:**
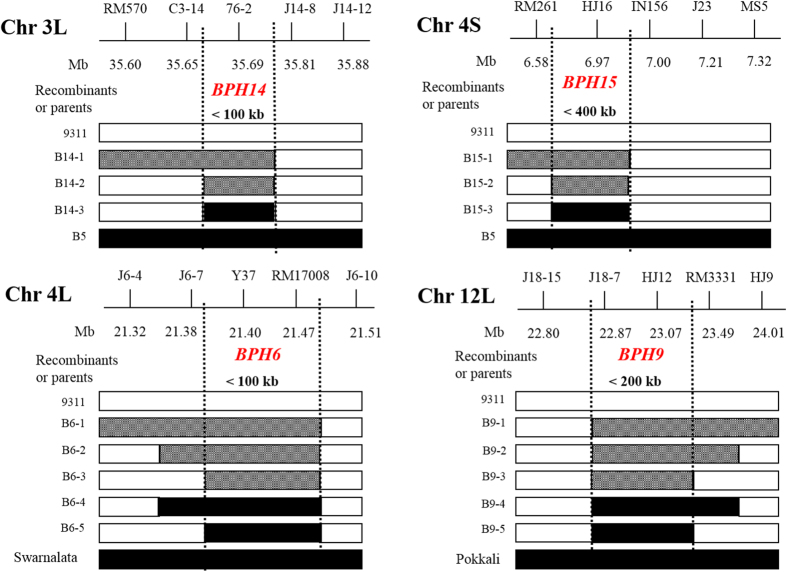
Positive and negative selection for *Bph14*, *Bph15*, *Bph6* and *Bph9*. The black and blank bars represent plants with marker genotype of homozygous donors and 9311, respectively. The grid bar represents heterozygous marker genotype.

Among the 13 genes, four (*Bph14*, *Bph15*, *Bph6* and *Bph9*) were transferred by positive and negative selection (Fig. 2). Taking *Bph6* as an example, closely linked markers Y37 and RM17008 were used for positive selection during the introgression process. Two other markers, J6-7 and J6-10, approximately 28 kb upstream of Y37 and 32 kb downstream of RM17008, respectively, were used in negative selection. Among 2,000 BC_1_F_2_ progenies (segregating at the *Bph6* locus), by selection on one side of the *Bph6* locus, one plant heterozygous in the region near the *Bph6* allele and homozygous for the 9311 region at the J6-7 locus, was selected and then backcrossed to 9311 to produce the BC_2_F_1_. Then five selected plants heterozygous at *Bph6* were selfed to produce a BC_2_F_2_ population. Among 3,000 BC_2_F_2_ progenies, two plants heterozygous for *Bph6*, and homozygous for the 9311 allele at the J6-10 locus, were selected. Finally, the linked segments around *Bph14*, *Bph15*, *Bph6* and *Bph9* were narrowed to less than 100 kb, 400 kb, 100 kb and 200 kb, respectively (Fig. 2).

For the remaining genes, flanking marker pairs closely linked to the target genes were used in positive selection, including XC4-9/IN15-6 for *Bph17*, RM586/RM589 for *Bph3*, XC3-14/IN76-2 for *QBph3*, XY4-17/XC4-27 for *QBph4*, XY4-17/HJ28 for *Bph20*, HJ12/HJ9 for *Bph21*, XC12-2/HJ12 for *Bph10*, HJ12/XC18-7 for *Bph18,* and J22/16717 for *Bph24* (Supplementary Table S1).

### Background selection

**Figure 3:**
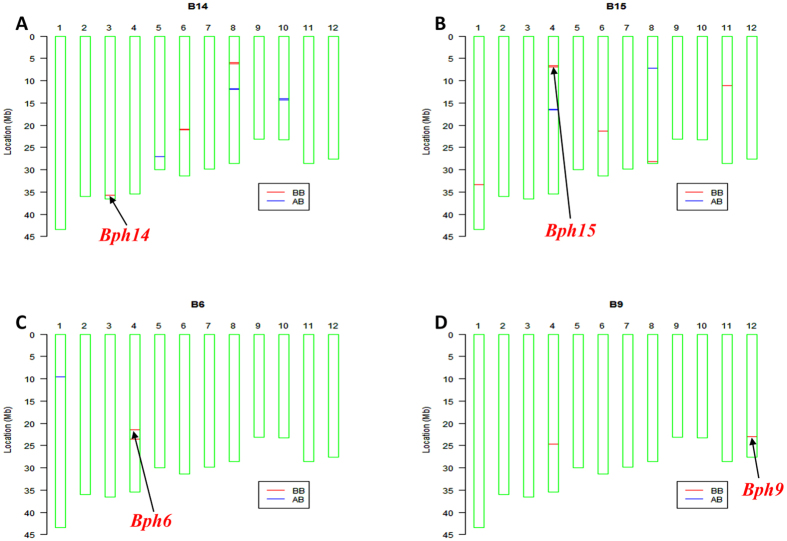
Haplotype maps of NILs carrying *Bph14* (**A**), *Bph15* (**B**), *Bph6* (**C**) and *Bph9* (**D**) analysed by the RICE6K array. BB genotype marked by the red bar is homozygous for the selected gene from the donor parent, AB genotype marked by the blue bar is heterozygous.

The selected recombinants B14-2, B15-2, B6-3 and B9-3 heterozygous at the *Bph14*, *Bph15*, *Bph6* and *Bph9* loci, respectively, were each backcrossed to 9311 to produce advanced backcross (BC) generation materials. In the BC_3_ to BC_4_ generations, the 6 K SNP chip^30^ was used to select individuals with the highest RPG. Selected individuals were self-pollinated to produce the BC_4_F_2_ generations. As shown in Fig. 3 individuals B14, B15, B6 and B9 not only had small donor segments linked to the target genes, but also carried few additional donor segments on other chromosomes. RPG recoveries were 99.7, 99.2, 99.8 and 99.9% in the monogenic NILs carrying *Bph14*, *Bph15*, *Bph6* and *Bph9*, respectively (Table 1). The other 9 genes were likewise, transferred by MABB using positive selection and background selection; RPG recoveries were 98.5, 92.9, 93.2, 98.5, 95.3, 98.4, 98.8, 97.8 and 97.5% in the NILs harboring *Bph17*, *Bph3*, *QBph3*, *QBph4*, *Bph10*, *Bph18*, *Bph20*, *Bph21* and *Bph24,* respectively (Supplementary Fig. S1, Table 1). Finally, 13 monogenic NILs were selected and designated as *Bph14*-NIL, *QBph3*-NIL, *QBph4*-NIL, *Bph17*-NIL, *Bph15*-NIL, *Bph20*-NIL, *Bph24*-NIL, *Bph6*-NIL, *Bph3*-NIL, *Bph9*-NIL, *Bph10*-NIL, *Bph18*-NIL and *Bph21*-NIL.

### Agronomic performance of NILs and their hybrids

A number of agronomic traits were measured, including days to heading (DTH), plant height (PH), panicle number (PN), number of grains (NG), number of grains per panicle (NPG), spikelet fertility (SF), 1000-grain weight (GW), and yield per plant (YD). *Bph3*-NIL and *Bph21*-NIL showed significant decreases in GW compared to 9311, *QBph3*-NIL had significantly higher SF and GW, resulting in increased yield (Table 1). The improved hybrids H2613S/*QBph4*-NIL and H2613S/*Bph15*-NIL had significantly higher SF compared to the conventional hybrid H2613S/9311. H2613S/*Bph24*-NIL showed significantly higher NG and NPG, but lower GW, leading to an equal yield compared to the conventional hybrid (Table 2). However, for most traits comparisons, there were no significant differences between the NILs and 9311, improved hybrids and conventional hybrids (Tables 1 and 2).

### Seedling response

**Figure 4:**
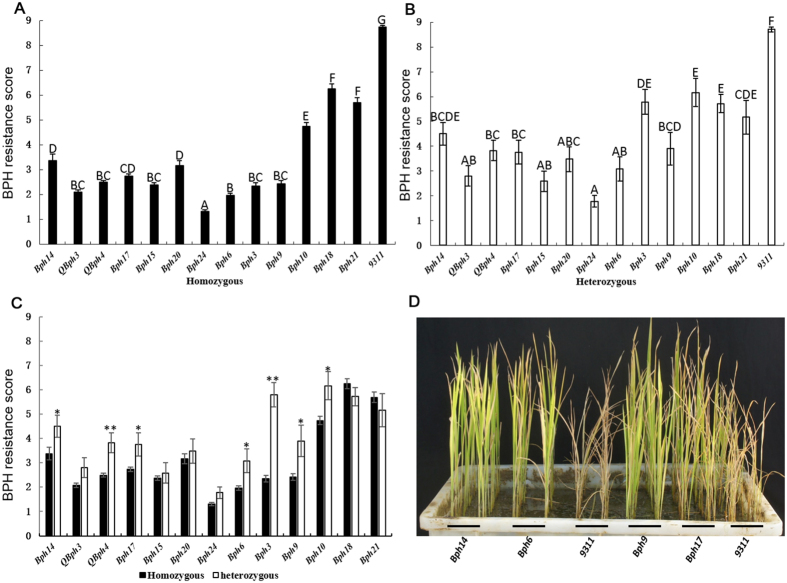
Seedling responses of NILs and their hybrids. (**A** and **B**) Response scores of NILs and their hybrids, respectively. (**C**) Comparison of the responses of NILs and corresponding hybrids. (**D**) Representative images of *Bph14*-NIL, *Bph6*-NIL, *Bph9*-NIL and *Bph17*-NIL damaged by BPH infestation. The sample sizes of each NIL and hybrid were 48 and 24, respectively. Uppercase letters above the error bars indicate significantly differences in ranking by Duncan’s test at *P* < 0.01. Error bars, SEM. * and ** significantly different at *P* < 0.05 and *P* < 0.01, respectively.

After about 14 days of BPH infestation in the greenhouse the 9311 control showed 100% wilting whereas the NILs were surviving. In short, the NILs harboring single genes or QTL on chromosome 4 (*QBph4*, *Bph17*, *Bph15*, *Bph20*, *Bph24* and *Bph6*) had higher resistance than those on chromosome 12 (*Bph10*, *Bph18* and *Bph21*), except for *Bph9* (Fig. 4A). The *Bph24*-NIL had the lowest response score (1.3), representing the highest level of seedling resistance among the 13 NILs. To explore the potential usefulness of these genes in hybrids, 13 monogenic hybrids between H2613S and the NILs were also evaluated. The hybrid response closely paralleled those of corresponding NILs (Fig. 4B). Hybrids carrying *Bph14*, *QBph4*, *Bph17*, *Bph6*, *Bph3*, *Bph9* and *Bph10* showed lower levels of resistance than the corresponding NILs, indicating incomplete dominance of these genes. However, there were no significant differences between the response scores of the remaining six monogenic NILs and their hybrids, indicating complete dominance of those genes (Fig. 4C).

### Honeydew excretion and survival rate of BPH on NILs

**Figure 5:**
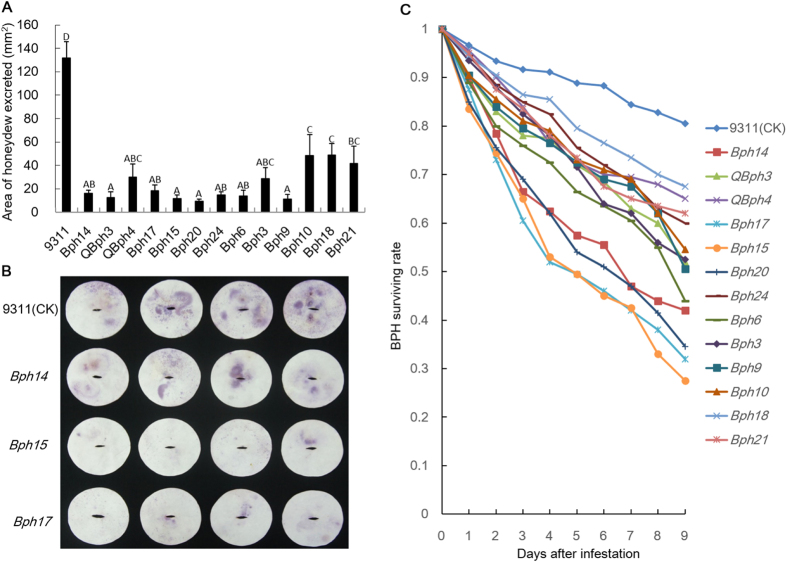
Areas of honeydew deposition (**A** and **B**) and survival rates of BPH on NILs (**C**). Uppercase letters above the error bars in A indicate significant differences in ranking by Duncan’s multiple range test at *P* < 0.01. Error bars, SEM. Tests were conducted in eight replications.

To determine whether the presence of resistance genes affected BPH growth and development, we compared the areas of honeydew excretion for BPH feeding on each NIL. The results were in accordance with the seedling responses (Fig. 5A,B), suggesting that honeydew deposition is a simple measurable indicator of BPH fitness on the lines. The excretion areas were grouped into four classes: the smallest, *QBph3*, *Bph9, Bph15 and Bph20* (approximate area 10 mm^2^); small, *QBph4, Bph3*, *Bph6*, *Bph14*, *Bph17* and *Bph24* (20 mm^2^); higher, *Bph10*, *Bph18*, and *Bph21* (50 mm^2^); the highest, 9311 (130 mm^2^) (Fig. 5A).

To test whether antibiosis was a factor in BPH resistance, BPH survival rates were measured on the monogenic NILs every day for 9 days after infestation (DAI). Mean BPH survival rates were lowest on *Bph15*-NIL, *Bph17*-NIL, *Bph20*-NIL and *Bph14*-NIL, and decreased more rapidly over the 9 days than on the other NILs (survival rate at 9 DAI: 28–43%) (Fig. 5C). Survival rates decreased less quickly on *Bph6*-NIL, *Bph9*-NIL, *Bph3*-NIL, *QBph3*-NIL and *Bph10*-NIL (45–55% at 9 DAI), and least slowly on *Bph18*-NIL, *QBph4*-NIL, *Bph21*-NIL and *Bph24*-NIL (65–75% at 9 DAI). The average survival rate on the control (9311) showed the slowest reduction (83% of the BPHs were alive at 9 DAI).

BPH survival rates on the NILs mostly paralleled the data for honeydew accumulation and seedling response. The lower effectiveness of the NILs with *QBph4* and *Bph24* compared to those with *Bph17, Bph15* and *Bph20* suggests these two genes might have different mechanisms of resistance.

### Sequence comparison of genes in three clusters

**Figure 6:**
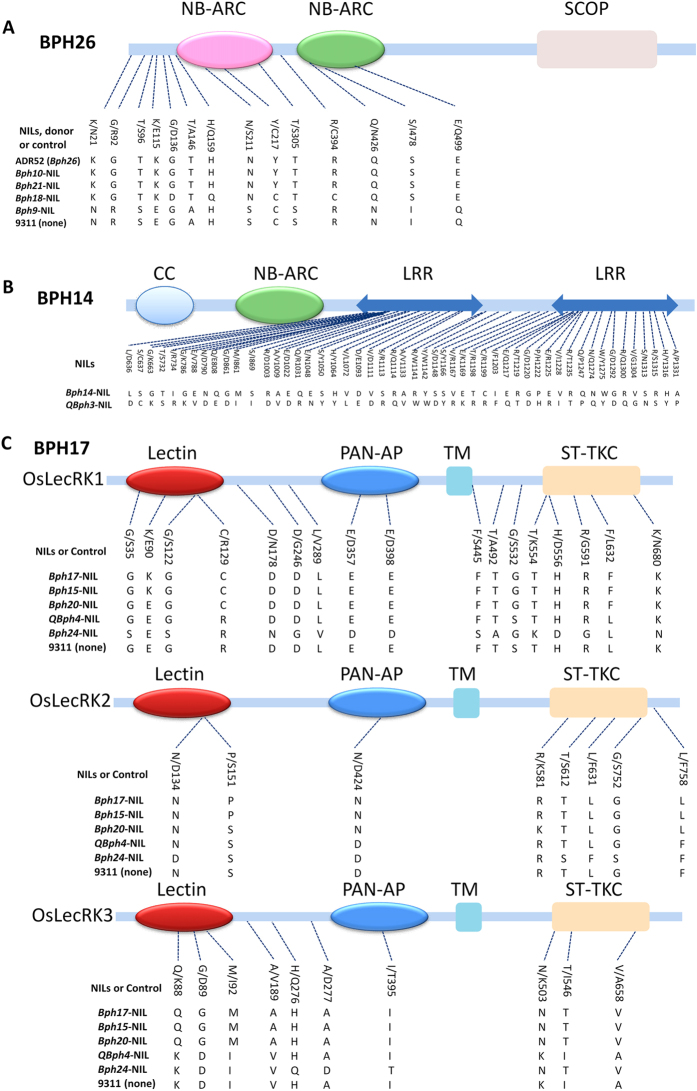
Sequence comparison of conserved amino acid sequences of cloned genes. (**A**) Comparison of the genes/QTLs on chromosome12 with *Bph26*. The gene sequence of *Bph18* was reported in Ji *et al*.^12^. (**B**) Comparison of *QBph3* and *Bph14*. (**C**) Comparison of the genes/QTLs on chromosome 4 with *Bph17*. The sequence information for 9311 is from the GenBank database (http://rise.genomics.org.cn/rice/index2.jsp).

Genes for BPH resistance in rice were reported to cluster on chromosomes 3, 4 and 12, among which *Bph14* on chromosome 3, *Bph17* on chromosome 4, *Bph29* on chromosome 6, and *Bph26* and *Bph18* on chromosome 12 have been cloned^8^,^11^,^12^,^13^,^14^. To explore whether the genes in the clusters surrounding *Bph14*, *Bph26* and *Bph17* were the same, we sequenced and compared the amino acid sequence of the *Bph26* alleles from *Bph9*-NIL*, Bph10*-NIL and *Bph21*-NIL, the *Bph14* allele from the *QBph3*-NIL and *Bph14*-NIL, and the *Bph17* allele from the *QBph4*-NIL, *Bph15*-NIL, *Bph17*-NIL, *Bph20*-NIL and *Bph24*-NIL. The proteins encoded by *Bph26* alleles from the monogenic NILs carrying *Bph10* and *Bph21* were identical in amino acid sequence as the cloned *Bph26*. *Bph18* and *Bph26* are different alleles with many amino acid substitutions^12^. However, due to inability to obtain the PCR product of the third exon of the *Bph26* allele from the *Bph9*-NIL using six pairs of specific primers, only the first and second exons were compared and a few nucleotide polymorphisms causing amino acid substitutions were detected (Fig. 6A). Compared to *Bph14,* the *Bph14* allele from *QBph3*-NIL had a number of amino acid substitutions in the LRR domain (Fig. 6B). Compared to the amino acid sequence of the cloned *Bph17*, that from *Bph15*-NIL was the same, while that from NILs of *QBph4*, *Bph20* and *Bph24* showed several substitutions (Fig. 6C).

## Discussion

To achieve improvement in target traits by MABB, breeders aim to minimize the introgressed segments from donors in order to reduce linkage drag, and to maximize reconstitution of recurrent parent genomes. Previously, improved lines contained large fragments (>1,000 kb) of the target gene regions, due to lack of suitable closely linked molecular markers and limited knowledge of the actual chromosomal locations of the resistance genes^14^. To reduce linkage drag, we performed positive and negative selection to obtain resistant recombinants between flanking markers in target regions based on high resolution physical maps of BPH resistance genes in two large backcross populations (BC_1_F_2_ and BC_2_F_2_). Introgressed segments containing four genes (*Bph14*, *Bph15*, *Bph6* and *Bph9*) were finally narrowed to less than 400 kb (Fig. 2). In contrast, the introgressed segments for the remaining nine genes exceeded 1,000 kb as negative selection was not employed (Supplementary Fig. S1).

In previous MABB programs, background selection was based on RFLP and SSR markers which had low resolution and inadequate whole genome coverage^15^,^21^. With development of next generation sequencing technology, large numbers of SNPs became available, and two breeding chips RICE6K and RiceSNP50 with high-throughput SNP arrays were developed in China^30^,^31^, making efficient whole-genome background selection a reality. Background selection with high resolution SNP markers was used in rice breeding to improve blast resistance and wide compatibility^16^,^32^. In the present study, the breeding chip RICE6K was employed in background selection for improving BPH resistance. Undesirable donor segments in each NIL could be viewed on the haplotype map produced by the chip. For example, after backcrossing and background selection, *Bph6*-NIL and *Bph9*-NIL only had four and three short segments from donors, and the RPG recoveries were 99.8 and 99.9%, respectively (Fig. 3). Our work demonstrated that positive and negative selection of target loci and whole genome background selection based on the 6 K SNP chip was a powerful way of developing monogenic NILs.

To date, eight BPH resistance genes, *Bph1*, *bph2*, *Bph3*, *Bph6*, *Bph14*, *Bph15*, *Bph18* and *Bph27 (t)* have been individually incorporated into *indica* or *japonica* varieties by MABB^18^,^19^,^20^,^21^,^33^,^34^,^35^. These NILs or ILs carrying single resistance genes have been reported as resistant to one or more BPH biotypes predominating in various countries. However, the real effects of these genes cannot be compared using the original source genotypes due to the diverse genetic backgrounds in which additional resistance QTLs may be present. In the present study, 13 BPH resistance genes were separately introduced into cv. 9311 by MABB. Generally, the NILs carrying *QBph4*, *Bph15*, *Bph17*, *Bph20*, *Bph24* and *Bph6* on chromosome 4 showed significantly higher resistance than those carrying *Bph10*, *Bph18* and *Bph21* on chromosome 12, indicating that the gene cluster on chromosome 4 was more effective in conferring BPH resistance. The response scores of most NILs consistently matched seedling response, honeydew deposition area, and BPH survival rate scores, indicating a similar resistance mechanism. However, the *Bph24*-NIL showed the highest level of seedling resistance and the least honeydew excretion, but a higher BPH survival rate, indicated that *Bph24* might mediate a resistance mechanism than antibiosis (Figs 4 and 5). However, the RPG coverage differs among the 13 NILs and this may have some effect on the agronomic performance and BPH responses (Table 1 and Supplementary Fig. S1). Previously, Hu *et al*.^18^ proved that improved hybrid rice containing *Bph14* and *Bph15* showed enhanced resistance compared to a conventional line. In our study, hybrid F_1_ descendants of H2613S and NILs showed higher resistance than conventional hybrid rice that lacked resistance genes. *Bph14*, *QBph4*, *Bph17*, *Bph6*, *Bph3*, *Bph9* and *Bph10* showed significantly less resistance in hybrids than corresponding NILs, indicating incomplete dominance (Fig. 4).

It is noteworthy that multiple BPH resistance genes cluster together on rice chromosomes; eight genes (*Bph1*, *bph2*, *Bph9*, *Bph10*, *Bph18*, *Bph19*, *Bph21* and *Bph26*) are clustered on chromosome 12 L, and six (*QBph4*, *Bph15*, *Bph12*, *Bph17*, *Bph20* and *Bph24*) on chromosome 4S^36^. These gene clusters might involve different genes, different alleles at a single locus, or even the same gene with different haplotypes^21^. Based on response phenotypes of the NILs and comparisons of amino acid sequence with the cloned *Bph26*, *Bph14* and *Bph17*, some aspects were resolved. The amino acid sequences of *Bph10* and *Bph21* were identical to *Bph26* and the response phenotypes of the two monogenic NILs were similar to that of *Bph26-*NIL, whereas *Bph9* and *Bph18* were different (Figs 4, 5 and 6). We inferred that *Bph10*, *Bph21* and *Bph26* might be the same gene, but *Bph9* and *Bph18* were likely different alleles in this locus. *QBph3* has a number of amino acid substitutions compared with *Bph14.* Hu *et al*.^22^ reported that *QBph3* and *Bph14* were tightly linked on chromosome 3 L, but the *QBph3*-NIL showed a higher degree of resistance than *Bph14*-NIL. Thus, they might be alleles or linked genes that mediate different resistance mechanisms. *Bph15* shared the same amino acid sequence as *Bph17*, and the *Bph15*-NIL had a similar BPH response phenotype to the *Bph17*-NIL with resistance scores of 2.4 versus 2.7, honeydew deposition area of 11.8 vs 18.1, and survival rates 33% vs 38%. However, there were differences between *Bph17* and the alleles from the *QBph4*, *Bph20* and *Bph24* NILs in amino acid sequence and response phenotype. These results indicated that *Bph15* was likely to be identical to *Bph17*, whereas *QBph4*, *Bph20* and *Bph24* might be different. Lv *et al*.^35^ and Hu *et al*.^22^ reported that *Bph15* and *QBph4* were located proximally to *Bph17*, thus differing from the present findings. One possible reason is that besides *Bph17* there is another resistance gene/QTL in the donor B5.

Cv. 9311 is an elite restorer line for two-line hybrid rice and HL CMS three-line hybrid rice because of its good adaptation, ideal plant type, good grain quality and high yield potential. However, due to the absence of disease (bacterial blight, blast) and insect (stem borer, BPH) resistance, the commercial application of 9311 is limited. Our 9311 NILs with high BPH resistance will provide a choice of parent lines for use in producing hybrid rice.

Generally pyramiding major resistance genes would enhance the resistance level of rice plant. However, whether the pyramided resistances will also improve the durability of BPH resistance is still unknown^37^. Furthermore, gene pyramiding might increase the risk of linkage drag, and higher resistance levels might lead to stronger selection pressure on BPH, thus accelerating evolution of the pest and resulting in failure of the resistance genes. In order to improve the durability of BPH resistance, 10 BPH resistance genes were transferred into 9311 individually and the corresponding multiline hybrid combinations developed in this study could be a possible strategy to prolong their effectiveness. Moreover, since these genes are from different *indica* cultivars and wild relatives with different resistance mechanisms, they may suppress the development of a harmful dominant race.

## Methods

### Plant materials and insects

Nine donor parents (DPs) were used to develop a set of monogenic NILs carrying genes for BPH resistance in the genetic background of cultivar (cv.) 9311. The DPs [RH (*Bph3* and *Bph17*), B5 (*Bph14* and *Bph15*), IR54751-1-2-44 (*QBph3* and *QBph4*), IR65482-4-136 (*Bph10*), IR71033-121 (*Bph20* and *Bph21*), IR65482-7-216 (*Bph18*), Swarnalata (*Bph6*), Pokkali (*Bph9*) and BR96 (*Bph24*)] were obtained from IRRI, Wuhan University and Guangxi Academy of Agricultural Sciences. Hua 2613S (H2613S) is an improved two-line thermo-sensitive genic male sterile line with blast resistance gene *R6* added by molecular marker assisted selection to the genetic background of Guangzhan 63S (data not shown). Both cv. 9311 and Guangzhan 63S are leading male and female parents for a number of commonly used two line hybrids in China, including Yangliangyou 6, the most widely cultivated hybrid in central and southern China during the last five years.

The BPH insects (predominantly biotype-2) used for infestation were collected from rice fields in Wuhan, and maintained on TN1 plants under greenhouse conditions at Huazhong Agricultural University.

### DNA extraction and marker analysis

Genomic DNA was extracted from fresh seedling leaves by the CTAB method^38^ with minor modifications. The PCR protocol was as follows: 2 μl (20 ng/μl) DNA in an 8 μl reaction mixture [2.0 μl 10 × buffer, 1.6 μl dNTP (2 mM), 1.4 μl MgCl_2_ (25 mM), 0.4 μl each primer (50 ng/μl), 2 μl ddH_2_O, and 0.2 μl Taq E (5 U/μl)], 10 μl ddH_2_O, and 20 μl mineral oil. The cycling regime was: 94 °C for 4 min, followed by 32 cycles of 94 °C/30 s, 55 °C/30 s, and 72 °C/40 s, and a final extension at 72 °C for 7 min. PCR products were separated on 4% non-denaturing polyacrylamide gels and detected by silver staining. SSR sequences were identified in Gramene (www.gramene.org), and Indel markers were designed based on sequence alignments of the Nipponbare and 9311 reference genomes in Rice Var Map (http://ricevarmap.ncpgr.cn/) (Table 1). For genome-wide genotyping based on the RICE6K SNP array, DNA amplification, fragmentation, chip hybridization, single base extension, staining and scanning were conducted by the Life Science and Technology Center, China National Seed Co. LTD, Wuhan, China^30^.

### Markers used for positive and negative selection

Positive selection for the presence of genes *Bph17*, *Bph3*, *QBph3*, *QBph4*, *Bph6*, *Bph9*, *Bph10*, *Bph14*, *Bph15*, *Bph18*, *Bph20*, *Bph21* and *Bph24* was conducted using gene-linked marker pairs XC4-9/IN15-6, RM586/RM589, C3-14/IN76-2, XY4-17/XC4-27, Y37/RM17008, J18-7/HJ12, XC12-2/HJ12, C3-14/IN76-2, RM261/HJ16, HJ12/J18-7, XY4-17/HJ28, HJ12/HJ9, XC12-2/HJ12 and HJ22/RM16717, respectively. Flanking marker pairs for negative selection were J64/J6-7/6-10 (*Bph6*), RM570/J14-8/J14-12 (*Bph14*), IN15-6/J23/MS5 (*Bph15*) and J18-15/RM3331/HJ9 (*Bph9*) (Supplemental Table S1).

### Collection of agronomic trait data from field experiments

The 26 NILs and hybrids containing the BPH resistance genes were planted in a randomized complete block design at Wuhan in the summer of 2015. Plots of each line consisted of two rows with 10 plants per row at a planting density of 17 cm between plants in a row and 27 cm between rows. The central eight plants from each plot, were used to measure agronomic traits including plant height (PH), days to heading (DTH), panicle number (PN), number of grains (NG), number of grains per panicle (NPG), spikelet fertility (SF), 1,000 grain weight (GW), and yield per plant (YD). There were three replications for each NIL and hybrid combination.

### Seedling response assays

The BPH bioassay was performed as a modified bulk seedling test in the greenhouse, following the method of Pathak *et al*.^39^. Seeds of 9311 and test lines were sown as random groups in 50 cm × 30 cm × 10 cm (height) plastic trays. Seedlings were thinned to 12 plants per line at the three-leaf stage and infested with second and third instar nymphs at a density of 15 insects per seedling. When all 9311 seedlings (control) had died [10–12 days after infestation (DAI)] the plants in other lines were examined, and each seedling was given a score of 1, 3, 5, 7 or 9 according to the criteria described by Huang *et al*.^40^ where higher scores indicate greater susceptibility to BPH. These tests were performed in three replications.

### Honeydew area

Determination of areas of honeydew deposition followed the method of Du *et al*.^11^. Thirty-day-old NILs and 9311 (control) were covered by inverted transparent plastic cups placed over a filter paper resting on plastic Petri dishes. After starving for 2 h, five fifth instar BPH nymphs were placed in each cup. Two days later, the filter papers were oven dried for 30 min at 60 °C and treated with 0.1% solution of ninhydrin in acetone. Areas of ninhydrin-positive deposits were measured using Image J software. Tests were conducted in eight replications.

### BPH survival rates

Survival rates were calculated following the method of Du *et al*.^11^. To determine nymph survival rates on rice lines, each plant was infested with 20 second and third instar nymphs and covered with a cylindrical plastic cup. Survival rates calculated as percentages of surviving nymphs divided by the total number of nymphs released at the beginning were recorded daily for 9 days.

### Statistical analysis

Mean phenotypic values of plants were compared using one-way ANOVA. Duncan’s multiple range and t tests were used for multiple mean comparisons. All statistical analyses were conducted using SPSS 7 for Windows version 16.0 (SPSS Inc., USA).

## Additional Information

**How to cite this article**: Xiao, C. *et al*. Development and evaluation of near-isogenic lines for brown planthopper resistance in rice cv. 9311. *Sci. Rep.* **6**, 38159; doi: 10.1038/srep38159 (2016).

**Publisher's note:** Springer Nature remains neutral with regard to jurisdictional claims in published maps and institutional affiliations.

## Supplementary Material

Supplementary Information 1

## Figures and Tables

**Table 1:** Measurements of agronomic traits of the NILs and recurrent parent 9311 in Wuhan in 2015.

NIL	DTH	PH (cm)	NG (g)	PN	NPG (g)	SF (%)	GW (g)	YD (g)	RPG (%)
*Bph14*-NIL	95.0 ± 0.8	120.3 ± 1.8	1569.9 ± 82.1	8.6 ± 0.4	182.4 ± 3.2	91.3 ± 0.4	31.5 ± 0.3	48.1 ± 2.7	99.7
*QBph3*-NIL	92.7 ± 0.6	123.5 ± 2.3	1682.0 ± 51.5	8.9 ± 0.3	188.4 ± 2.0	94.2 ± 0.5^*^	33.6 ± 0.2^**^	55.7 ± 1.7^*^	93.2
*QBph4*-NIL	93.7 ± 0.6	122.1 ± 1.5	1611.5 ± 75.5	8.8 ± 0.3	183.7 ± 3.0	93.3 ± 0.3	31.4 ± 0.2	49.5 ± 2.2	98.5
*Bph17*-NIL	96.0 ± 2.0	124.0 ± 2.9	1603.3 ± 50.5	8.9 ± 0.2	179.1 ± 2.3	91.4 ± 0.5	30.7 ± 0.2	48.2 ± 1.6	98.5
*Bph15*-NIL	92.7 ± 1.2	125.6 ± 3.0	1652.3 ± 77.6	8.7 ± 0.4	188.9 ± 3.9	92.1 ± 0.2	31.4 ± 0.2	50.9 ± 2.3	99.2
*Bph20*-NIL	96.0 ± 1.2	125.9 ± 3.2	1629.9 ± 63.9	8.6 ± 0.3	188.2 ± 2.4	91.6 ± 0.3	31.0 ± 0.1	49.7 ± 2.1	98.8
*Bph24*-NIL	93.0 ± 1.7	122.3 ± 2.0	1703.0 ± 61.3	9.0 ± 0.3	191.9 ± 6.4	91.7 ± 0.4	31.8 ± 0.8	50.6 ± 1.7	97.5
*Bph6*-NIL	94.0 ± 0.6	124.4 ± 1.9	1710.3 ± 73.6	9.3 ± 0.4	184.7 ± 3.2	91.8 ± 0.3	31.4 ± 0.2	52.0 ± 2.2	99.8
*Bph3*-NIL	93.0 ± 0.6	122.5 ± 2.0	1638.2 ± 70.8	8.9 ± 0.3	184.5 ± 2.4	94.2 ± 0.5	29.1 ± 0.2^**^	46.6 ± 1.8^*^	92.9
*Bph9*-NIL	94.7 ± 1.2	124.2 ± 1.3	1619.2 ± 48.5	8.9 ± 0.3	181.1 ± 1.8	92.6 ± 0.5	31.4 ± 0.2	50.1 ± 1.3	99.9
*Bph10*-NIL	95.0 ± 1.0	119.7 ± 1.9	1671.3 ± 76.0	8.7 ± 0.4	191.3 ± 2.1	92.2 ± 0.5	30.9 ± 0.3	50.4 ± 2.2	95.3
*Bph18*-NIL	94.0 ± 0.6	122.8 ± 1.6	1593.8 ± 52.8	8.8 ± 0.3	180.6 ± 2.1	91.3 ± 0.4	31.8 ± 0.2	49.4 ± 1.7	98.4
*Bph21-*NIL	96.0 ± 2.0	123.9 ± 1.9	1758.8 ± 60.5	9.3 ± 0.3	190.0 ± 2.3	92.2 ± 0.7	29.3 ± 0.2^**^	50.3 ± 1.8	97.8
9311 (CK)	94.0 ± 0.6	121.4 ± 1.8	1670.5 ± 48.7	9.0 ± 0.2	185.3 ± 3.0	92.0 ± 0.3	31.0 ± 0.2	50.9 ± 1.5	100

DTH: days to heading, PH: plant height, NG: number of grains per plant, PN: panicle number, NPG: number of spikelet per panicle, SF: spikelet fertility, GW: 1000-grain weight, YD: yield per plant, RPG: recurrent parent genome. *, **, significantly different from 9311 at *P* = 0.05 and *P* = 0.01, respectively.

**Table 2:** Measurements of agronomic traits of hybrids of 2613 S and the NILs in Wuhan, 2015.

Hybrid	DTH	PH (cm)	NG (g)	PN	NPG (g)	SF (%)	GW (g)	YD (g)
S/*Bph14*-NIL	94.7 ± 1.2	129.9 ± 2.1	1774.8 ± 50.8	10.5 ± 0.2	168.2 ± 2.0	82.7 ± 1.1	29.3 ± 0.2	50.8 ± 1.5
S/*QBph3*-NIL	94.0 ± 2.0	129.2 ± 1.2	1777.8 ± 100.3	10.5 ± 0.3	167.8 ± 4.1	84.4 ± 0.7	29.7 ± 0.2	51.7 ± 3.1
S/*QBph4*-NIL	94.0 ± 0.0	128.1 ± 1.5	1777.1 ± 70.3	10.3 ± 0.2	170.6 ± 3.3	88.3 ± 0.8^**^	29.7 ± 0.3	51.1 ± 2.0
S/*Bph17*-NIL	93.7 ± 0.6	130.5 ± 1.6	1751.4 ± 55.3	10.5 ± 0.2	165.4 ± 2.1	83.1 ± 1.0	29.0 ± 0.3	50.0 ± 1.7
S/*Bph15*-NIL	92.7 ± 1.2	126.6 ± 3.0	1792.1 ± 63.2	10.6 ± 0.2	168.2 ± 3.6	87.2 ± 1.9^*^	29.4 ± 0.5	51.9 ± 2.0
S/*Bph20*-NIL	92.3 ± 0.6	130.9 ± 1.6	1749.4 ± 91.0	10.4 ± 0.3	167.5 ± 4.3	83.5 ± 0.7	28.7 ± 0.5	48.9 ± 2.4
S/*Bph24*-NIL	93.0 ± 1.0	128.3 ± 2.0	1959.1 ± 103.8^*^	10.7 ± 0.6	184.9 ± 6.2^*^	85.6 ± 1.0	28.4 ± 0.3^*^	54.5 ± 2.8
S/*Bph6*-NIL	94.3 ± 1.5	127.4 ± 2.6	1851.7 ± 79.5	10.8 ± 0.3	170.4 ± 3.9	83.7 ± 0.7	29.5 ± 0.2	53.9 ± 2.6
S/*Bph3*-NIL	92.0 ± 1.7	127.4 ± 2.0	1756.0 ± 90.9	10.4 ± 0.2	167.9 ± 3.9	83.4 ± 3.1	29.4 ± 0.3	50.9 ± 2.6
S/*Bph9*-NIL	92.3 ± 1.5	127.9 ± 1.7	1804.2 ± 98.1	10.6 ± 0.3	170.7 ± 3.7	83.7 ± 0.7	29.5 ± 0.3	52.5 ± 2.6
S/*Bph10*-NIL	94.7 ± 1.2	127.4 ± 1.6	1757.2 ± 64.3	10.5 ± 0.3	166.9 ± 2.6	84.0 ± 0.7	29.6 ± 0.3	50.8 ± 1.8
S/*Bph18*-NIL	96.0 ± 1.0	127.8 ± 1.5	1722.1 ± 90.2	9.8 ± 0.4	169.3 ± 7.4	84.0 ± 0.9	29.6 ± 0.2	49.6 ± 2.6
S/*Bph21*-NIL	96.0 ± 1.0	128.8 ± 2.3	1745.8 ± 42.4	10.2 ± 0.2	164.2 ± 2.6	83.1 ± 0.9	29.3 ± 0.2	50.0 ± 1.2
2613S/9311 (CK)	94.7 ± 1.2	128.9 ± 2.1	1759.1 ± 50.3	10.2 ± 0.5	168.1 ± 5.7	84.0 ± 0.5	29.2 ± 0.2	51.0 ± 1.8

DTH: days to heading, PH: plant height, NG: number of grains per plant, PN: panicle number, NPG: number of spikelet per panicle, SF: spikelet fertility, GW: 1000-grain weight, YD: yield per plant, RPG: recurrent parent genome. *, **, significantly different from 9311 at *P* = 0.05 and *P* = 0.01, respectively.
