# Supplementary material for: Development and evaluation of near-isogenic lines for brown planthopper resistance in rice cv. 9311
Source: Sci Rep. 2016 Nov 30;6:38159. doi: 10.1038/srep38159 (PMC5128867; doi:10.1038/srep38159)
Supplement: Supplementary Information 1 [file srep38159-s1.pdf]

Development, evaluation and sequencing of near-isogenic lines for brown planthopper resistance in rice cv. 9311

Cong Xiao[1], Jie Hu[1], Yi-Ting Ao[1], Ming-Xing Cheng[1], Guan-Jun Gao[1], Qing-Lu Zhang[1],

Guang-Cun He[2] & Yu-Qing He[1]*

[1]National Key Laboratory of Crop Genetic Improvement, Huazhong Agricultural University, Wuhan 430070, China. [2]State Key Laboratory of Hybrid Rice, College of Life Sciences, Wuhan University, Wuhan 430070, China.

*Correspondence: yqhe@mail.hzau.edu.cn.

National Key Laboratory of Crop Genetic Improvement and National Center of Crop Molecular Breeding, Huazhong Agricultural University, Wuhan 430070, China

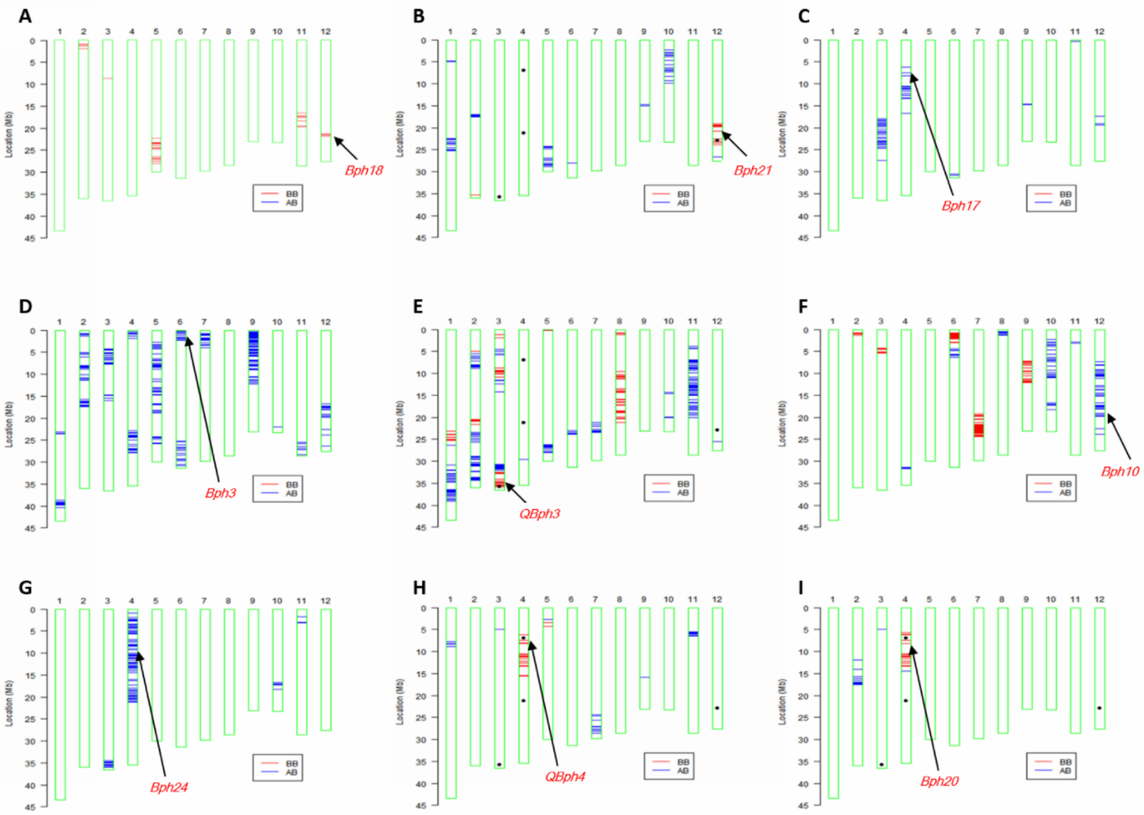

Supplementary Figure S1. Haplotype maps of nine NILs carrying *Bph18* (A), *Bph21* (B), *Bph17* (C), *Bph3* (D), *QBph3* (E), *Bph10* (F), *Bph24* (G), *QBph4* (H) and *Bph20* (I) tested by the RICE6K array.

Supplementary Table S1. Markers and primers used in the study

Markers or primers[a]	Marker type	chr	Location[b]	Forward primer(5'-3')	Reverse primer(5'-3')
RM570	SSR	3	35595750	GTTCTTCAACTCCCAGTGCG	TGACGATGTGGAAGAGCAAG
C3-14	SSR	3	35654007	GGCAAAATTAGACGGCACG	GAATATGCATTTTGTTTGGAG
IN76-2	InDel	3	35689799	CTGCTGCTGCTCTCGTATTG	CAGGGAAGCTCCAAGAACAG
J14-8	InDel	3	35809723	CAACATTTGGATGCATTGC	CTTTATTATCTACGGCAC
J14-12	InDel	3	35883380	GGGGACGATTTGGTAACTAG	CTATTAATGTAAAATATCACGTG
XY4-17	InDel	4	6574764	ACACGCAATCGACTACTAG	ATAGAAGCCAATACACACC
RM261	SSR	4	6579056	CTACTTCTCCCCTTGTGTCG	TGTACCATCGCCAAATCTCC
XC4-27	InDel	4	6899436	GCATAAGCGCCCTAGCC	GCTAGTTGCAGGCACGC
XC4-9	InDel	4	6937039	AATGGCCAAGGATTGATGC	TTTCTTATCGACTCATAGG
HJ16	InDel	4	6937072	CACATGTATTTGATCGAG	GGGATTCAGGAGTAGCGGG
IN15-6	InDel	4	7006594	AGGTGAAGCTGATGTGCTTG	CGATACTTATTGCAACACAC
J23	InDel	4	7224671	GTACTTCAAAACTGTCAG	CTTATGTGTAATGTTTTCACCC
MS5	InDel	4	7251959	TTGTGGGTCCTCATCTCCTC	TGACAACTTGTGCAAGATCAAA
HJ28	InDel	4	8239137	CTAACGATGACGACTAC	GTCAGCGGCAAATCCTG
HJ22	InDel	4	15267615	CTATGTGGTCCCATTTCTTC	GTGTCGGTTCACATGCTCC
RM16717	SSR	4	15474930	ACAGCACTCATCATTCCATGTCC	GATAACTGATCGACGCGACACC
J6-4	InDel	4	21320230	CCCTCCATGTCAGACGC	GTTGTGCGGATGCCTGAC
J6-7	InDel	4	21381048	GCCACGAGTCCAGGCGC	GGAGGATTTCGGACGATGCG
Y37	InDel	4	21409939	TCGACCTACGGCTTTTCTGTA	TAGATTCCTTCGGTTCCCATT
RM17008	SSR	4	21476894	TTACCTTCGATTAGCTGCTGTTGC	ATTCCTTGCATTACAGACGGTAGC
J6-10	InDel	4	21508709	CAACACGATCAACTCACCTAG	CGCAACCTCGCTCATGC
RM589	SSR	6	1381886	ATCATGGTCGGTGGCTTAAC	CAGGTTCCAACCAGACACTG
RM586	SSR	6	1477833	ACCTCGCGTTATTAGGTACCC	GAGATACGCCAACGAGATACC
XC12-2	InDel	12	19521625	GCCTTCGCTTCACATGGG	TTTAACCCGGTAAAGAATG
J18-15	InDel	12	22648024	CTTTTACCAGAAACGTGCC	CTCGGTTTTCGAGAGCATG
J18-7	InDel	12	22875241	GACCCCCTTCGAGTCTAAGAAC	CTTCTTTGAACTCATAGACAG
HJ12	InDel	12	23074750	CAAAATCCCGGTGTAAGC	CTTGATGTCTGAAGACCTAG
RM3331	SSR	12	23494476	CCTCCTCCATGAGCTAATGC	AGGAGGAGCGGATTTCTCTC
HJ9	InDel	12	23873868	CAGTTGGAGTCCACCTCG	CTTGCTTCGGAAGCGATGAATG
BPH14-LRR-14		3		ATGGTATTAAGTAAACTGC	AGTCATCGTTAATATCAGG
BPH14-LRR-47		3		AGGAGACTTTGGTTCTGG	TACACTGCGTACGAAGACC
BPH17-OsLecRK1		4		ATTGTTTACTCCAATAACGGT	TTATGGGACAGAGTGAGTATTT
BPH17-OsLecRK2		4		CTTTCGCAGGGTGGCAAATAGGGT	CCTTCGCTGCTCACTAGGACCGTGTA
BPH17-OsLecRK3		4		GCCCGCTCGCTACCGCT	CGGTCCTGCACATTACTGTAG
BPH26-Exson1		12		TAGCATCAGTCCCTTGCTTGTTTGC	ATTGATTTAATTAGCAGACAAGTTG
BPH26-Exson2		12		AACTCTCGTCTCGTCTTAAAATATA	AGTAGTAATGTGCGTAGCAATGGAG
BPH26-Exson3		12		CTAGTGCCAGTTACTCCGATAAATAT	TATGCACTAGCATCACTACA

Primers for *Bph17* and *Bph26* were reported in Liu *et al.*[14] and Tamura *et al.*[13].

Location data are based on version 7.0 of Nipponbare sequence from MSU
